# The Protect Effects of Chitosan Oligosaccharides on Intestinal Integrity by Regulating Oxidative Status and Inflammation under Oxidative Stress

**DOI:** 10.3390/md19020057

**Published:** 2021-01-25

**Authors:** Ruixia Lan, Qingqing Chang, Linlin Wei, Zhihui Zhao

**Affiliations:** College of Coastal Agriculture Sciences, Guangdong Ocean University, Zhanjiang 524088, China; Lanrx@gdou.edu.cn (R.L.); changqingqing@outlook.com (Q.C.); wllgdhy@163.com (L.W.)

**Keywords:** chitosan oligosaccharides, inflammation cytokines, intestine, oxidative status, oxidative stress

## Abstract

The aim of this study was to evaluate the effects of the dietary supplementation of chitosan oligosaccharides (COS) on intestinal integrity, oxidative status, and the inflammation response with hydrogen peroxide (H_2_O_2_) challenge. In total, 30 rats were randomly assigned to three groups with 10 replications: CON group, basal diet; AS group, basal diet + 0.1% H_2_O_2_ in drinking water; ASC group, basal diet + 200 mg/kg COS + 0.1% H_2_O_2_ in drinking water. The results indicated that COS upregulated (*p* < 0.05) villus height (VH) of the small intestine, duodenum, and ileum; mucosal glutathione peroxidase activity; jejunum and ileum mucosal total antioxidant capacity; duodenum and ileum mucosal interleukin (IL)-6 level; jejunum mucosal tumor necrosis factor (TNF)-α level; duodenum and ileum mucosal IL-10 level; the mRNA expression level of zonula occludens (ZO)-1 in the jejunum and ileum, claudin in the duodenum, nuclear factor-erythroid 2-like 2 in the jejunum, and heme oxygenase-1 in the duodenum and ileum; and the protein expression of ZO-1 and claudin in jejunum; however, it downregulated (*p* < 0.05) serum diamine oxidase activity and D-lactate level; small intestine mucosal malondialdehyde content; duodenum and ileum mucosal IL-6 level; jejunum mucosal TNF-α level; and the mRNA expression of IL-6 in the duodenum and jejunum, and TNF-α in the jejunum and ileum. These results suggested COS could maintain intestinal integrity under oxidative stress by modulating the intestinal oxidative status and release of inflammatory cytokines.

## 1. Introduction

The pernicious effects of oxidative stress on intestinal function have been widely studied [1,2]. Studies have demonstrated oxidative stress was one of the vital factors contributing to intestinal injury and disfunction [3,4]. Oxidative stress was frequently related to increases in interleukin (IL)-1β, IL-6, and tumor necrosis factor (TNF)-α [3,5], and undermined intestinal function [6]. Both oxidative stress and inflammation could disturb intestinal function by over-production of reactive oxygen species (ROS) and pro-inflammatory cytokines [2,7]. Impaired intestinal integrity, accompanied with intestinal permeability and histological changes [8,9,10], promote the transportation of toxic luminal substances, which may contribute to intestinal disease and even death [10,11,12]. The intestine predominantly responds to virous stressors, especially oxidative stress and inflammation [3,13,14,15]. Hence, alleviating intestinal oxidative stress and inflammation are important in maintaining intestinal function. Therefore, an ideal candidate, which has free-radical scavenging activity, and antioxidant and anti-inflammation capacities, is urgently needed to maintain intestinal function under oxidative stress.

Chitosan oligosaccharides (COS) are the degraded products of chitosan; compared to chitosan, COS are non-toxic, non-allergenic, less viscous, and entirely soluble in water [16]. COS also have multiple properties, including free-radical scavenging, and antioxidant, anti-inflammatory, antibacterial, and immune-enhancing activities, which capture much attention, and they are widely used in biomedical medicine and agriculture science [17]. Qiao et al. [18] reported COS could relieve sepsis by virtue of their antioxidation property and anti-inflammatory effect. Liu et al. [19] demonstrated COS could remit oxidative damage in umbilical vein endothelial cells. Lan et al. [20] demonstrated COS remitted H_2_O_2_-induced oxidative stress in the liver, kidney and spleen by alleviating oxidative and inflammation stress. Furthermore, COS also have positive effects on intestinal health [21,22]. It is believed that intestinal oxidative stress and the inflammation response are highly related to intestinal integrity and function. COS captured attention due to antioxidant and anti-inflammatory activities. Therefore, this study was done to evaluate the effects of dietary COS supplementation on intestinal integrity, oxidative status, and the inflammatory response with H_2_O_2_ challenge.

## 2. Results

### 2.1. Intestinal Mucosal Morphology

**Figure 1:**
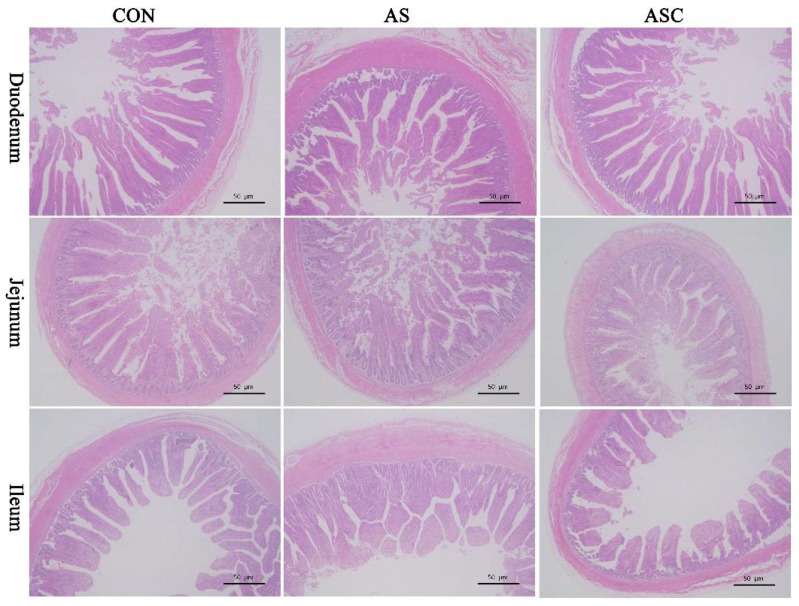
Effects of chitosan oligosaccharides on intestinal morphology when challenged with hydrogen peroxide. CON group, basal diet; AS group, basal diet + 0.1% hydrogen peroxide (H_2_O_2_) in drinking water; ASC group, basal diet + 200 mg/kg COS + 0.1% H_2_O_2_ in drinking water. Scale label: 50 μm.

Figure 1 and Table 1 show intestinal morphology indices. Compared with the CON group, the villus height (VH) and VH to crypt depth (CD) ratio of the small intestine were decreased (*p* < 0.05) in the AS group (basal diet + 0.1% H_2_O_2_ in drinking water). Compared with the AS group, COS increased the VH of the small intestine, and the VH:CD of the duodenum and jejunum.

### 2.2. Intestinal Permeability

**Figure 2:**
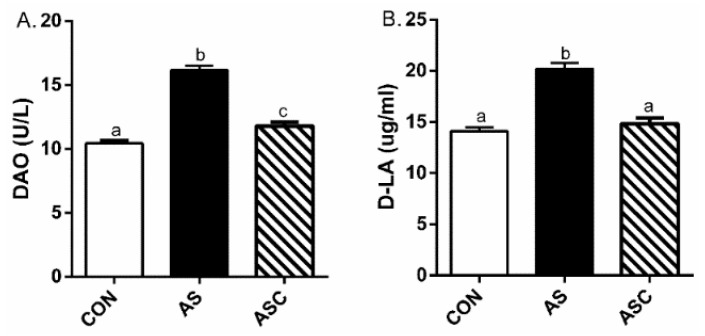
Effects of chitosan oligosaccharides on serum DAO and D-LA after hydrogen peroxide challenge. CON group, basal diet; AS group, basal diet + 0.1% H_2_O_2_ in drinking water; ASC, basal diet + 200 mg/kg COS + 0.1% H_2_O_2_ in drinking water. (**A**) DAO content; (**B**) D-LA activity. Values are means ± standard errors. Columns that have different numbers above them statistically differ (*p* < 0.05). ^a,b,c^ Columns that have different letter statistically differ (*p* < 0.05).

Compared with the CON group, serum diamine oxidase (DAO) activity and d-lactate acid (D-LA) content were upregulated (*p* < 0.05) in the AS group (Figure 2). Compared with the AS group, COS downregulated (*p* < 0.05) serum DAO activity and D-LA content.

### 2.3. Antioxidant Capacity

**Figure 3:**
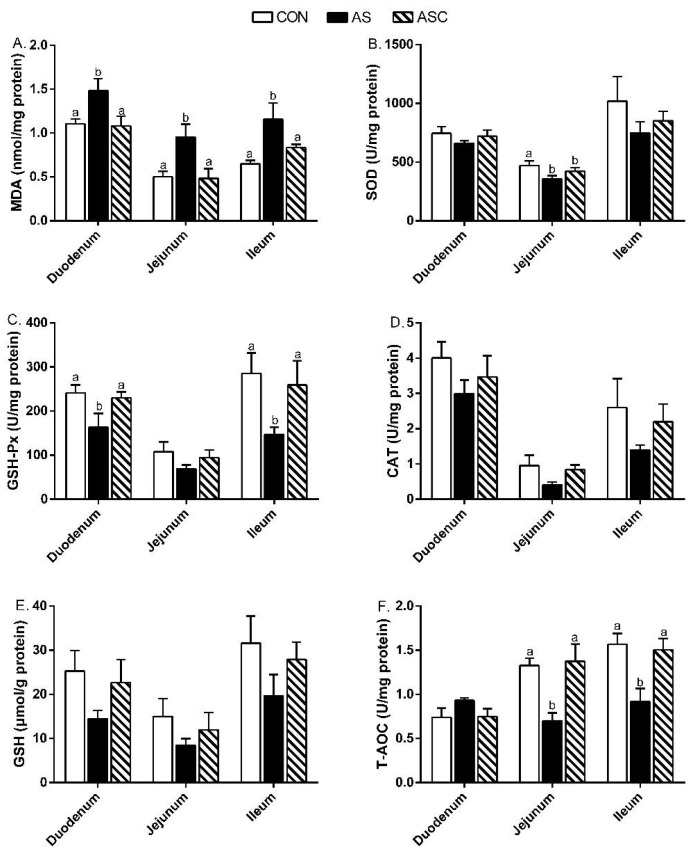
Effects of chitosan oligosaccharides on small intestine antioxidant indicators when challenged with hydrogen peroxide. CON group, basal diet; AS group, basal diet + 0.1% H_2_O_2_ in drinking water; ASC, basal diet + 200 mg/kg COS + 0.1% H_2_O_2_ in drinking water. (**A**) MDA content; (**B**) SOD activity; (**C**) GSH-Px activity; (**D**) CAT activity; (**E**) GSH content; (**F**) T-AOC activity. Values indicate means ± standard errors. ^a,b^ Columns that have different letter statistically differ (*p* < 0.05).

Figure 3 presents the antioxidant indicators in the small intestinal mucosa. In duodenum, the malondialdehyde (MDA) content in the AS group was higher (*p* < 0.05) than the contents in the CON and ASC groups, but the glutathione peroxidase (GSH-Px) activity was lower (*p* < 0.05). In the jejunum, the MDA content in the AS group was higher (*p* < 0.05) than in the CON and ASC groups; the superoxide dismutase (SOD) activity in the CON group was higher (*p* < 0.05) than in the AS and ASC groups; the total antioxidant capacity (T-AOC) activity in the AS group was lower (*p* < 0.05) than in the CON and ASC groups. In the ileum, the MDA contents in the CON and ASC groups were lower (*p* < 0.05) than that in the AS group, but the GSH-Px and T-AOC activities were higher.

### 2.4. Free Radical Scavenging Activity

**Figure 4:**
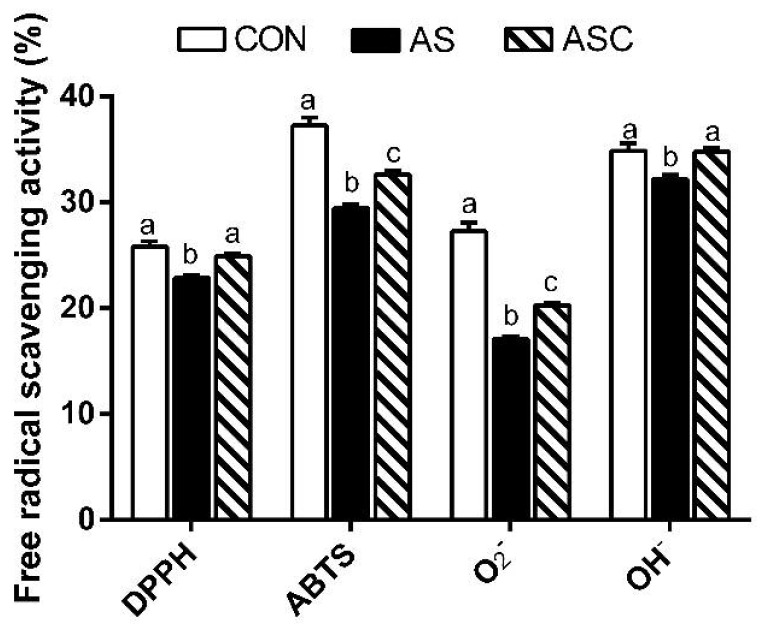
Effects of chitosan oligosaccharides on the free-radical scavenging activity of the jejunum when challenged with hydrogen peroxide. CON group, basal diet; AS group, basal diet + 0.1% H_2_O_2_ in drinking water; ASC group, basal diet + 200 mg/kg COS + 0.1% H_2_O_2_ in drinking water. The free-radical scavenging activity was calculated on the basis of the protein content (mg/mL) of the jejunum mucosa. Values indicate means ± standard errors. ^a,b,c^ Columns that have different letter statistically differ (*p* < 0.05).

Figure 4 presents the free-radical scavenging capacities in jejunum mucosa. The 1,1-diphenyl-2-picrylhydrazyl (DPPH), 2,2′-azino-bis-3-ethylbenzothiazoline-6-sulfonic acid (ABTS), O_2_^−^ (superoxide radical) and hydroxyl radical (OH^−^) scavenging activities in the CON and ASC groups were higher (*p* < 0.05) than in the AS group.

### 2.5. Intestinal Mucosal Immunity

**Figure 5:**
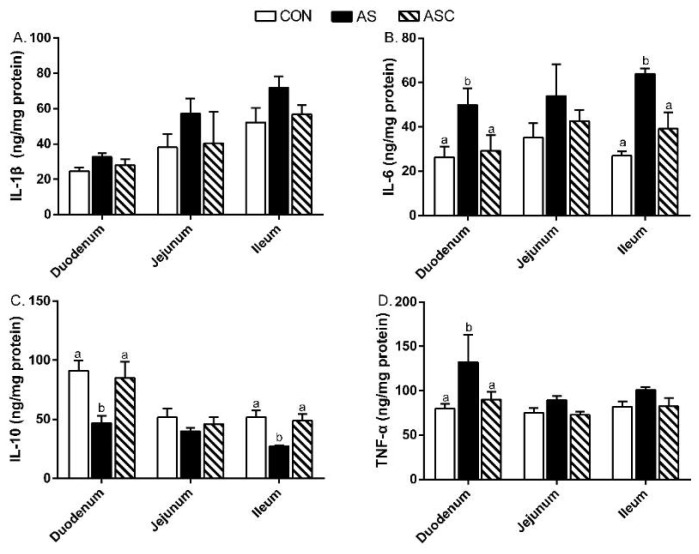
Effects of chitosan oligosaccharides on inflammatory cytokines in the small intestine when challenged with hydrogen peroxide. CON group, basal diet; AS group, basal diet + 0.1% H_2_O_2_ in drinking water; ASC group, basal diet + 200 mg/kg COS + 0.1% H_2_O_2_ in drinking water. (**A**) IL-1β content; (**B**) IL-6 content; (**C**) IL-10 content; (**D**) TNF-α content. Values are indicated as means ± standard errors. ^a,b^ Columns that have different letter statistically differ (*p* < 0.05).

In the duodenum, the IL-6 and TNF-α contents in the AS group were enhanced (*p* < 0.05) compared to CON and ASC groups (Figure 5), but the IL-10 content was decreased (*p* < 0.05). In the ileum, the IL-6 content in the AS group was increased (*p* < 0.05) over CON and ASC groups, but the IL-10 content was decreased (*p* < 0.05).

**Figure 6:**
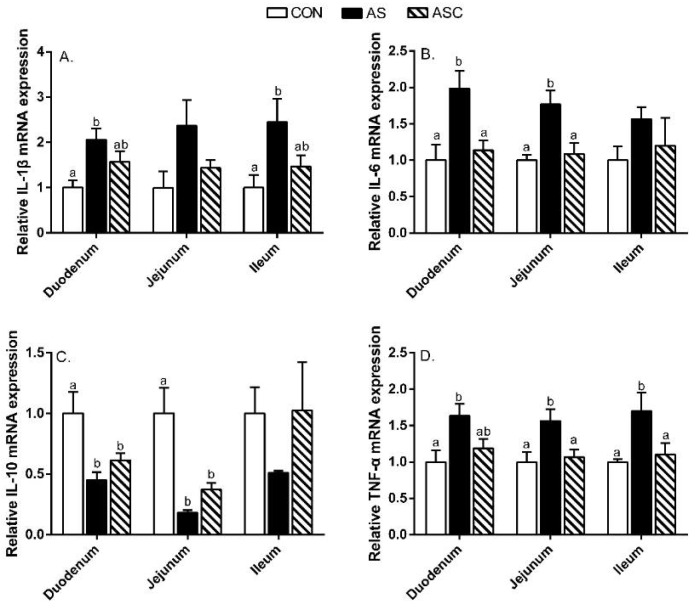
Effects of chitosan oligosaccharides on inflammatory cytokine-related gene mRNA expression in the small intestine when challenged with hydrogen peroxide. CON group, basal diet; AS group, basal diet + 0.1% H_2_O_2_ in drinking water; ASC group, basal diet + 200 mg/kg COS + 0.1% H_2_O_2_ in drinking water. (**A**) relative IL-1β mRNA level; (**B**) relative IL-6 mRNA level; (**C**) relative IL-10 mRNA level; (**D**) relative TNF-α mRNA level. Values indicate means ± standard errors. ^a,b^ Columns that have different letter statistically differ (*p* < 0.05).

Furthermore, we also examined the related cytokines’ gene expression in the small intestine (Figure 6). In the duodenum, the relative gene expression levels of IL-1β and TNF-α in the AS group were higher (*p* < 0.05) than in the CON group; IL-6 in the AS group was enhanced (*p* < 0.05) over CON and ASC groups; IL-10 in the AS and ASC groups was less present (*p* < 0.05) compared to the CON group. In the jejunum, the relative gene expression levels of IL-6 and TNF-α in the AS group were increased (*p* < 0.05) compared to CON and ASC groups; IL-10 in the AS and ASC groups was decreased (*p* < 0.05) compared to the CON group. In the ileum, the relative gene expression of IL-1β in the AS group was enhanced (*p* < 0.05) compared to the CON group; TNF-α in the AS group was higher (*p* < 0.05) than in CON and ASC groups.

### 2.6. Intestinal Barrier Function-Related Gene Expression

**Figure 7:**
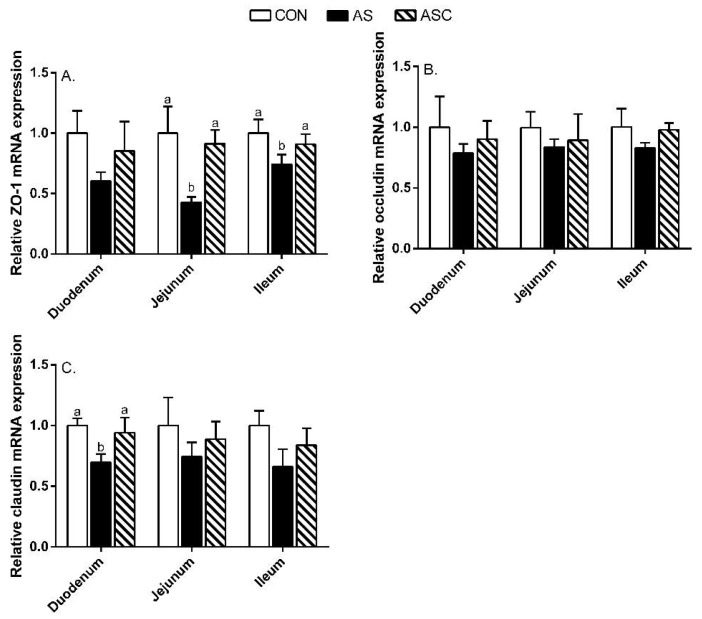
Effects of chitosan oligosaccharides on intestinal barrier-related gene mRNA expression of the small intestine when challenged with hydrogen peroxide. CON group, basal diet; AS group, basal diet + 0.1% H_2_O_2_ in drinking water; ASC group, basal diet + 200 mg/kg COS + 0.1% H_2_O_2_ in drinking water. (**A**) relative ZO-1 mRNA expression level; (**B**) relative occludin mRNA expression level; (**C**) relative claudin mRNA expression level. Values indicated as means ± standard errors. ^a,b^ Columns that have different letter statistically differ (*p* < 0.05).

**Figure 8:**
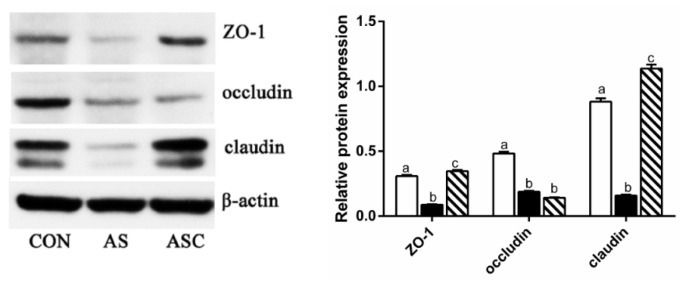
Effects of chitosan oligosaccharides on tight junction protein expression in the jejunum when challenged with hydrogen peroxide. CON group, basal diet; AS group, basal diet + 0.1% H_2_O_2_ in drinking water; ASC group, basal diet + 200 mg/kg COS + 0.1% H_2_O_2_ in drinking water. ^a,b,c^ Columns that have different letter statistically differ (*p* < 0.05).

In the duodenum, the relative gene expression of claudin in the AS group was decreased (*p* < 0.05) compared to CON and ASC groups (Figure 7). In the jejunum and ileum, the relative gene expression of zonula occludens (ZO)-1 in the AS group was decreased (*p* < 0.05) compared to CON and ASC groups. In addition, we found that the expression levels of ZO-1 and claudin protein in the AS group were decreased (*p* < 0.05) compared to CON and ASC groups (Figure 8); and the expression of occludin protein in the AS and ASC groups was decreased (*p* < 0.05) compared to the CON group in jejunum mucosa.

**Figure 9:**
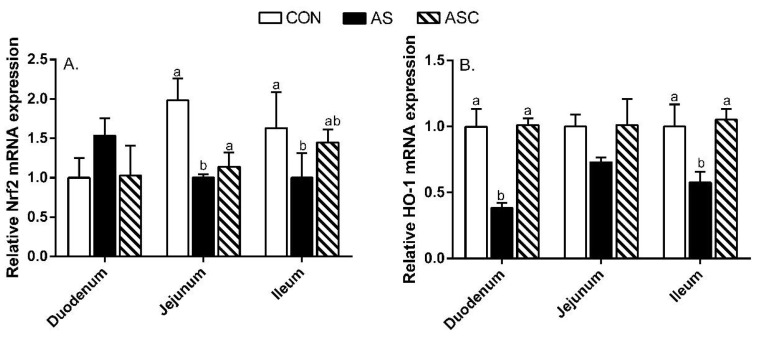
Effects of chitosan oligosaccharides on Nrf2 and HO-1 gene mRNA expression in the small intestine when challenged with hydrogen peroxide. CON group, basal diet; AS group, basal diet + 0.1% H_2_O_2_ in drinking water; ASC group, basal diet + 200 mg/kg COS + 0.1% H_2_O_2_ in drinking water. (**A**) relative Nrf2 mRNA expression level; (**B**) relative HO-1 mRNA expression level. Values indicated as means ± standard errors. ^a,b^ Columns that have different letter statistically differ (*p* < 0.05).

We also examined the critical gene expression in the antioxidant signaling pathway. As shown in Figure 9, in the duodenum, the relative gene expression of heme oxygenase (HO)-1 in the AS group was decreased (*p* < 0.05) compared to CON and ASC groups. In the jejunum, the relative gene expression of nuclear factor-erythroid 2-like 2 (Nrf2) in the AS group was lower (*p* < 0.05) than that in the CON group. In the ileum, the relative gene expression of Nrf2 in the AS group was decreased (*p* < 0.05) compared to the CON group; and the relative gene expression of HO-1 in the AS group was decreased (*p* < 0.05) compared to CON and ASC groups.

## 3. Discussion

Intestinal barrier integrity was commonly assessed by gut morphology, serum DAO activity, and D-LA activity. The gut morphology was a useful biomarker of the stress response of the intestinal tract [9,23,24]. The DAO activity and D-LA content were biomarkers of intestinal permeability [3,11,25]. In this study, oxidative stress decreased the VH of the small intestine, and enhanced DAO content and D-LA activity, demonstrating that oxidative stress resulted in intestinal injury by increasing intestinal permeability and decreasing VH [3,9]. As expected, COS decreased DAO content and D-LA activity, and increased the VH of the small intestine; hence, COS had positive effects on intestinal permeability. These results are also in line with former studies by Li et al. [21] and Zhao et al. [26], who indicated COS decreased serum DAO activity. The structure of the intestinal morphology reflected gut health status. Li et al. [21] illustrated COS increased the VH of the duodenum and ileum in broilers. Liu et al. [27] and Liu et al. [28] reported dietary COS supplementation increased the VH of the jejunum and ileum in weaning pigs. The positive effects of COS on intestinal permeability and morphology could explain the improving intestinal function with COS supplementation.

The tight junction proteins maintained and regulated the intestinal barrier function. The tight junction proteins mainly consisted of the transmembrane proteins claudin and occludin, and peripheral membrane protein ZO-1. Therefore, the decreased mRNA expression of ZO-1, claudin, and occludin reflected intestinal barrier dysfunction [4]. In this study, oxidative stress downregulated the protein expression of ZO-1, occludin, and claudin in the jejunum; and the mRNA expression of claudin in the duodenum and ZO-1 in the jejunum and ileum, which were all consistent with the results reported by Song et al. [29] and Cao et al. [1]. COS upregulated the protein expression of ZO-1 and claudin in the jejunum, and the mRNA expression of ZO-1 in the jejunum and ileum, and claudin in the duodenum—similarly to other studies on mice fed high-fat diets [30], dexamethasone-challenged broilers [31], and weaning pigs [32,33]. These results demonstrated that COS could alleviate oxidative-induced intestinal barrier function partly by maintaining the intestinal structure, intestinal permeability, and tight junction functionality.

Accumulating evidence indicates that oxidative status was an important factor in intestinal barrier function. An imbalance between oxidation and the antioxidant defense system leads to oxidative stress and inflammation, and finally, induces intestinal barrier dysfunction [12]. SOD, GSH-Px, and CAT were regarded as the main antioxidant enzymes for scavenging free radicals. GSH was regarded as the most important non-enzymatic antioxidant which scavenges single oxygen molecules and hydroxyl radicals. The T-AOC can reflect the total antioxidant capacity. MDA is an indicator of oxidative stress [34]. H_2_O_2_ can stimulate ROS over-production, disrupt the activity of antioxidant enzymes, and induce lipid peroxidation [35,36]. Consistently, in this study, oxidative stress induced higher small intestine MDA content, along with lower duodenum and ileum mucosal GSH-Px activity, jejunum mucosal SOD activity, and jejunum and ileum mucosal T-AOC activity. COS reduced duodenum, jejunum, and ileum mucosal MDA content; and increased duodenum and ileum mucosal GSH-Px activity, and jejunum and ileum mucosal T-AOC activity, suggesting COS could alleviate intestinal mucosal oxidative stress by improving the antioxidative enzyme activity and decreasing MDA content. These results are consistent with Lan et al. [20], who indicated COS could increase SOD, CAT, GSH-Px, and T-AOC activity, and decrease the MDA level with an H_2_O_2_ challenge. Li et al. [21] also indicated COS increased SOD activity in the duodenum’s mucosa and decrease the MDA level in the jejunum and ileum’s mucosa in broilers. Similarly, Li et al. [22] indicated that dietary COS supplementation increased the inhibition of hydroxy radical capacity, and GSH, T-AOC, GSH-Px, and SOD activity, whereas decreased MDA content in the ileum mucosa of broilers. Nrf2 is a nuclear transcription factor and plays a vital role in antagonizing oxidative stress [37]. Our results show decreased mRNA expression of Nrf2 in the jejunum and ileum, and HO-1 in the duodenum and ileum by H_2_O_2_ challenge. Other studies illustrated the increased Nrf2 mRNA expression level could increase the mRNA expression of SOD and GSH-Px [3]; the decreased Nrf2 and HO-1 mRNA expression levels may be related to a response to oxidative stress. As expected, COS enhanced the mRNA expression of Nrf2 in the jejunum and HO-1 in the duodenum and ileum—similarly to other studies on doxorubicin-challenged rats [38] and mice fed a high-fat diet [39]. Collectively, the combined results illustrate that the increased antioxidant enzyme activity may be mediated by Nrf2/HO-1 signaling pathway. Additionally, the efficiency of the free-radical scavenging capacities reflected the neutralization of free radical capacities, or hydrogen donor capacity [40]. In this study, COS improved the radical scavenging capacity of the jejunum mucosa; that may relate to antioxidant capacity and hydrogen donation ability [17].

Inflammation cytokines play vital roles in the inflammatory and immune responses. The accumulating literature illustrates inflammation is an important marker in intestinal disfunction [3,12]. Chen et al. [12] indicated that over-production of cytokines could change the intestinal permeability and tight junction structure by modulating tight junction-related genes expression in weaning piglets. The over-production of IL-1β, IL-6, and TNF-α directly resulted in intestinal mucosal injury [41,42]. Therefore, suppressing the over-production of intestinal mucosal IL-1β, IL-6, and TNF-α was a useful way to maintain the intestinal function. Previous studies indicated that stressors could disturb the balance between anti- and pro-inflammatory responses by increasing pro-inflammatory cytokines’ production [12,43,44]. In this study, the duodenum and ileum mucosal IL-6 content, and jejunum mucosal TNF-α level were higher, whereas the duodenum and ileum mucosal IL-10 levels were decreased, in the AS group compared to the CON group, indicating that oxidative stress resulted in inflammation in the intestine. Furthermore, the levels of mRNA expression of IL-1β in the duodenum and ileum, IL-6 in the duodenum and jejunum, and TNF-α in the small intestine in the AS group were increased compared to the CON group, but the expression levels of IL-10 in the duodenum and jejunum were decreased. Dietary COS supplementation decreased the duodenum and ileum mucosal IL-6 level and jejunum mucosal TNF-α level; inhibited the expression of IL-6 in the jejunum and ileum, and TNF-α in the jejunum and ileum; and increased the duodenum and ileum mucosal IL-10 levels, all of which was in consistent with the results of Hu et al. [33], who reported COS reduced IL-1β and TNF-α mRNA expression levels in jejunum mucosa in weaning pigs. Besides, COS decreased the IL-6 and TNF-α mRNA expression levels in the liver of mice fed a high-fat diet [39]. These results suggest that COS may alleviate intestinal inflammation by suppressing the levels of IL-1β, IL-6, and TNF-α [45,46]. However, in this study, we ignored the immune cells in the mucosal immune system, especially the mast cells, which play a vital role in the regulation of intestinal mucosal immune function and intestinal barrier function. Further study would focus on this point.

In conclusion, COS had beneficial effects on intestinal integrity by improving the antioxidant capacity and suppressing the release of inflammatory cytokines. Dietary COS supplementation may be an effective nutritional strategy to alleviate the detrimental effects of oxidative stress.

## 4. Materials and Methods

### 4.1. Animals, Diets, and Experimental Design

In total, 30 male Sprague–Dawley rats (8–10 weeks old, 178.39 ± 5.12 g) were purchased from Beijing Administration Office of Laboratory Animals and acclimatized for 7 days before the experiment. The rats were provided with a pelleted diet, had free access to diet and water, and were housed at constant temperature (24 ± 2 °C) and relative humidity (60% ± 5%) on a 12-h light–dark cycle. The basal diet composition is shown in Table 2. The experimental protocol and use of rats were approved by the Animal Care and Use Committee of Guangdong Ocean University, Zhanjiang, China (SYXK-2018-0147, 2018).

H_2_O_2_ induced oxidative stress by generation of potent ROS [23,24,25]. ROS caused lipid peroxidation, membrane disintegration, and endothelial cell damage [26]. The 30 rats were randomly divided into three groups: CON, basal diet; AS group, basal diet + 0.1% H_2_O_2_ in drinking water; ASC, basal diet + 200 mg/kg COS + 0.1% H_2_O_2_ in drinking water.

### 4.2. Sample Collection

At the end of the experiment, all rats were anesthetized and sacrificed to collected blood samples through the eyeballs. The blood samples were centrifuged at 4 °C, 3200× *g*, for 10 min to obtain serum for further analysis. The duodenum, jejunum, and ileum were divided into two parts, one part fixed in 10% buffered formalin for morphology analysis, the rest for collecting mucosa, and frozen in liquid nitrogen for further analysis.

### 4.3. Serum Diamine Oxidase (DAO) and d-Lactate Acid (D-LA)

Serum DAO activity and d-LA level were measured with commercial kits (Nanjing Jiancheng Institute of Bioengineering, Nanjing, China).

### 4.4. Intestinal Morphology

The intestinal morphology analysis was performed according our previously described methods [2].

### 4.5. Intestinal Antioxidant Parameters and Inflammatory Cytokines

About 1 g of each mucosal sample was homogenized at a ratio of 1:9 (weight/volume) with ice-cold PBS. Homogenate was centrifuged at 3200× *g* for 10 min at 4 °C to obtain supernatant; Bradford method was used to determine the supernatant protein concentration. The antioxidant parameters and inflammatory cytokines were measured with corresponding assay kits (Nanjing Jiancheng Bioengineering Institute, Nanjing, China).

### 4.6. Free Radical Scavenging Activities

DPPH, ABTS, O_2_^−^, and OH^−^ scavenging activities were evaluated by following the methods described in another study [20].

### 4.7. Gene Expression Analysis

Total RNA extraction, cDNA reverse transcription, and real-time polymerase chain reaction analysis were done according to our previous described methods [47]. The primers are shown in Table 3. The relative mRNA expression was calculated by 2^−∆∆Ct^ method.

### 4.8. Western Blot Analysis

The procedures of the Western blot assay were according to the description of Alhaithloul et al. [37].

### 4.9. Statistical Analysis

The individual rat was regarded as the experiment unit, SAS 2003 (version 9.1, SAS Institute Inc., Cary, NC, USA) was used to analyze the statistic. Duncan’s multiple range test was used to check the variance among the groups, and the differences were considered significant at *p* < 0.05.

## Figures and Tables

**Table 1:** Effects of chitosan oligosaccharides on intestinal mucosal morphology when challenged with hydrogen peroxide.

Items ^1^	CON	AS	ASC	SEM ^2^	*p*-Value
Duodenum					
VH (μm)	277.71 ^a^	219.07 ^b^	258.72 ^c^	5.70	<0.0001
CD (μm)	104.32	97.34	104.08	2.48	0.1265
VH:CD	2.67 ^a^	2.26 ^b^	2.50 ^a^	0.06	0.0021
Jejunum					
VH (μm)	209.73 ^a^	157.59 ^b^	190.96 ^a^	6.86	0.0010
CD (μm)	84.68	79.47	81.85	3.41	0.5751
VH:CD	2.48 ^a^	2.00 ^b^	2.34 ^a^	0.05	0.0002
Ileum					
VH (μm)	170.16 ^a^	116.56 ^b^	150.19 ^a^	7.26	0.0013
CD (μm)	67.69	61.19	65.81	2.41	0.1969
VH:CD	2.52 ^a^	1.91 ^b^	2.29 ^ab^	0.13	0.0221

^1^ COS, chitosan oligosaccharide; CON group, basal diet; AS group, basal diet + 0.1% hydrogen peroxide (H_2_O_2_) in drinking water; ASC group, basal diet + 200 mg/kg COS + 0.1% H_2_O_2_ in drinking water; VH, villus height; CD, crypt depth. ^2^ SEM, standard error of mean. ^a,b,c^ Two different superscripts indicate a significant difference in the same row (*p* < 0.05).

**Table 2:** The composition of basal diet.

Ingredients (g/kg)	Basal Diet	Basal Diet with 200 mg/kg COS
Cornstarch	464.00	463.80
Chitosan oligosaccharides	0	0.20
Casein	140.00	140.00
Dextrinized cornstarch	155.00	155.00
Sucrose	100.00	100.00
Soybean oil	40.00	40.00
Cellulose acetate	50.00	50.00
Mineral premix ^1^	35.00	35.00
Vitamin premix ^2^	10.00	10.00
l-Methionine	1.80	1.80
l-Cystine	1.80	1.80
Choline bitartrate	2.30	2.30
Tert-butylhydroquinone	0.10	0.10
Gross energy (MJ/kg)	16.22	16.20

^1^ Mineral premix (mg/kg of premix): CaCO_3_, 3.70 × 10^5^; KH_2_PO_4_, 1.96 × 10^5^; K_3_C_6_H_5_O_7_·H_2_O, 7.08 × 10^4^; NaCl, 7.4 × 10^4^; K_2_SO_4_, 4.66 × 10^4^; MgO, 2.4 × 10^4^; FeC_6_H_5_O_7_H_2_O, 6.06 × 10^3^; ZnCO_3_, 1.65 × 10^3^; MnCO_3_, 630; CuCO_3_, 324; NaSiO_3_·9H_2_O, 1.45 × 10^3^; CrK(SO_4_)·12H_2_O, 275; LiCl, 17.4; H_3_BO_3_, 81.5; NaF, 63.5; NiCO_3_·2Ni(OH)_2_·4H_2_O, 30.6; NH_4_VO_3_, 6.6; sucrose was added to make a total of 1 kg; ^2^ vitamin premix (mg/kg of premix): nicotinic, 3.0 × 10^3^; calcium pantothenate, 1.6 × 10^3^; pyridoxine hydrochloride, 700; thiamine hydrochloride, 600; riboflavin, 600; folic acid, 200; d-biotin, 20; cyanocobalamin, 2.5 × 10^3^; a-tocopherol, 1.5 × 10^4^; cholecalciferol, 250; phylloquinone, 75; sucrose was added to make a total of 1 kg.

**Table 3:** Primers for real-time PCR.

Gene	Accession NO.	Primer Sequence (5′ to 3′)	Product Size (bp)
GAPDH	NM_017008.4	F: GGCAAGTTCAACGGCACAG R: GACGCCAGTAGACTCCACGAC	144
IL-1β	NC_005102.4	F: CCACCTCCAGGGACAGGATA R: TGGGATCTACACTCTCCAGC	132
IL-6	NM_012589.2	F: CAAGTCCGGAGAGGAGACT R: TTCTGACAGTGCATCATCGC	172
IL-10	NM_012854.2	F: TGCGACGCTGTCATCGATTT R: GTAGATGCCGGGTGGTTCAA	186
TNF-α	NM_012675.3	F: ACACACGAGACGCTGAAGT R: TCCAGTGAGTTCCGAAAGCC	93
ZO-1	NM_001106266.1	F: GCCAGCTTTAAGCCTCCAGA R: TGGCTTCGCTTGAGGTTTCT	144
Occludin	NM_031329.2	F: GATCTAGAGCCTGGAGCAACG R: ATTGGGTTTGAATTCATCCGGC	166
Claudin-1	NM_031699.2	F: GCTGTCATCGGGGGCATAAT R: CCTGGCCAAATTCATACCTGG	136
Nrf2	NM_031789.2	F: TTTGTAGATGACCATGAGTCG R: TGTCCTGCTGTATGCTGCTT	142
HO-1	NM_012580.2	F: TTAAGCTGGTGATGGCCTCC R: GTGGGGCATAGACTGGGTTC	90

## Data Availability

The data presented in this study are available on request from the corresponding author.
